# Differences in the Inflammatory Response Induced by Acute Pancreatitis in Different White Adipose Tissue Sites in the Rat

**DOI:** 10.1371/journal.pone.0041933

**Published:** 2012-08-01

**Authors:** Sabrina Gea-Sorlí, Laia Bonjoch, Daniel Closa

**Affiliations:** Department of Experimental Pathology, IIBB-CSIC, IDIBAPS and CIBEREHD, Barcelona, Spain; University of Valencia, Spain

## Abstract

**Background:**

There is increasing evidence of the role of adipose tissue on the systemic effects of acute pancreatitis. Patients with higher body mass index have increased risk of local and systemic complications and patients with android fat distribution and higher waist circumference are at greater risk for developing the severe form of the disease. Here we evaluated the changes on different areas of adipose tissue and its involvement on the inflammatory response in an experimental model of acute pancreatitis.

**Methods:**

Pancreatitis was induced in male Wistar rats by intraductal administration of sodium taurocholate. Orlistat was administered to inhibit lipase activity. Activation of peritoneal macrophages was evaluated by measuring IL1β and TNFα expression. Inflammation was evaluated by measuring myeloperoxidase activity in mesenteric, epididymal and retroperitoneal areas of adipose tissue. Changes in the expression of inflammatory mediator in these areas of adipose tissue were also evaluated by RT-PCR.

**Results:**

Pancreatitis induces the activation of peritoneal macrophages and a strong inflammatory response in mesenteric and epididymal sites of adipose tissue. By contrast, no changes were found in retroperitoneal adipose tissue. Inhibition of lipase prevented the activation of macrophages and the local inflammation in adipose tissue.

**Conclusions:**

Our results confirm the involvement of adipose tissue on the progression of systemic inflammatory response during acute pancreatitis. However, there is a considerable diversity in different adipose tissue sites. These differences need to be taken into account in order to understand the progression from local pancreatic damage to systemic inflammation during acute pancreatitis.

## Introduction

Multisystemic organ failure is a frequent complication of severe acute pancreatitis. In addition of the initial inflammatory process in the pancreas, the release of cytokines and hydrolytic enzymes induces the propagation of inflammatory response to distant organs. The extrapancreatic effects of severe acute pancreatitis include systemic inflammation, disseminated fat necrosis, acute lung injury, renal failure and shock [1].

Obesity has long been recognized as a risk factor for a poor outcome in acute pancreatitis. A number of studies indicated that patients with higher body mass index have increased risk of local and systemic complications, and possibly death in acute pancreatitis [2]–[3]. Interestingly, fat distribution has also been found to be important and patients with android fat distribution and higher waist circumference are at greater risk for developing the severe form of the disease [4]–[5].

The correlation between body fat and the progression of acute pancreatitis results in a growing interest to understanding the changes occurring in white adipose tissue during acute pancreatitis. In this sense, disseminated fat necrosis is a characteristic feature that appears in the severe forms of the disease. It is considered a consequence of the release of lipolytic enzymes, mainly lipase and phospholipase, from damaged pancreatic acinar cells. These areas of visceral fat are sources of cytokines and adipokines but also of lipid mediators. In the lasts years, these lipid metabolites and, in particular, fatty acids generated by the action of lipase, emerge as relevant players on the progression of multisystem organ failure.

It has been reported that lipid extracts of fat necrosis substantially increases the activation of macrophages while lipid extract of control adipose tissue does not has this effect [6]. This effect seems to correlate with higher oxidative modifications of fatty acids. In this line, it has been recently demonstrated that the toxic effects of fatty acids released by peripancreatic adipose tissue contribute to increase pancreatic cell damage and also increase the expression of cytokines and chemokines [7]. These effects are linked to unsaturated fatty acids, which constitute more than 70% of total fatty acids present in triglycerides stored on adipose tissue. Finally, unsaturated fatty acids from necrotic adipose tissue show chemical modifications that increase its toxicity [8]. Myeloperoxidase activity is highly increased in necrotic adipose tissue due to the infiltration of neutrophiles. This enzyme catalyzes the chlorination of unsaturated fatty acids thus generating fatty acid chlorohydrins of oleic and linoleic acids that, in addition of its direct toxicity, have a number of pro-inflammatory effects on endothelial cells macrophages or epithelial cells.

However, different adipose tissue sites possess different characteristics, including cell size, proliferative responses and ability to react to hormonal and pharmacological stimuli [9]–[10]. These differences could be involved in the fact that not only body mass index but also the body fat distribution is important in the correlation between adipose tissue and severity of acute pancreatitis. In this work we examined the changes on different areas of adipose tissue and its involvement on the inflammatory response in an experimental model of acute pancreatitis.

## Results

**Figure 1:**
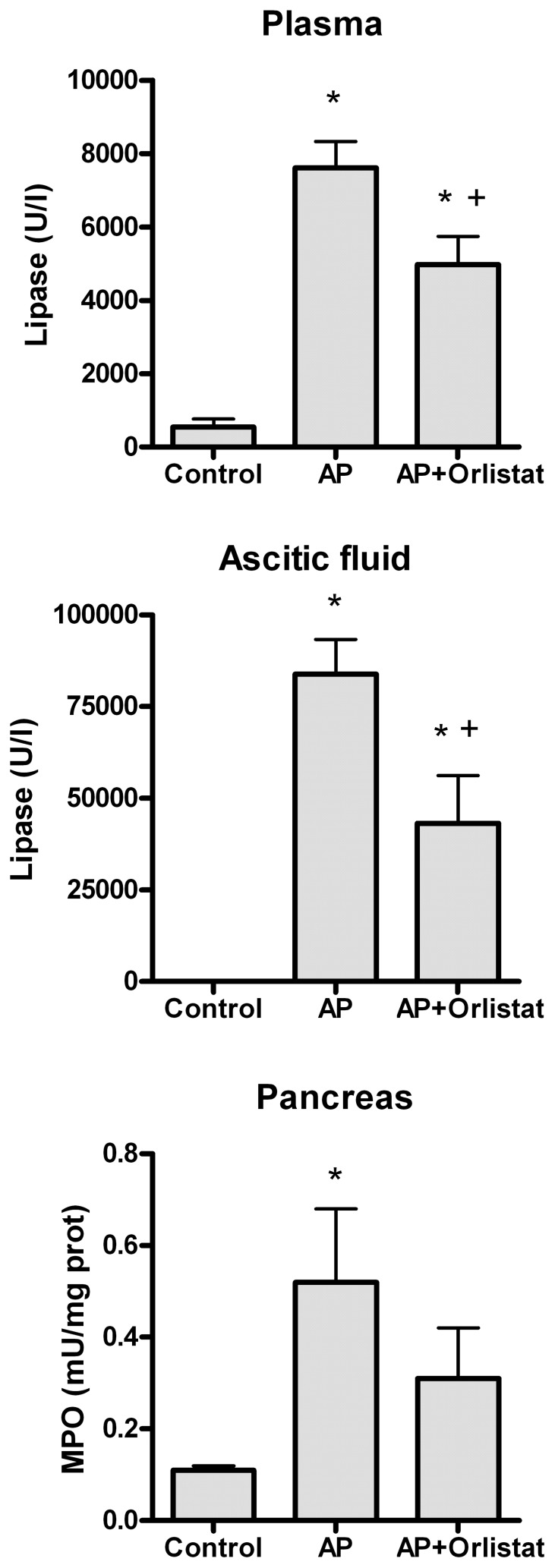
Acute pancreatitis (AP) results in significant increases in lipase activity in plasma and ascitic fluid as well as in myeloperoxidase (MPO) in pancreas. Orlistat treatment significantly inhibits lipase activity but also the increase in MPO activity in pancreas. *  =  p<0.05 vs control; +  =  p<0.05 vs AP.

Pancreatitis results in significant increases in lipase activity in plasma (Figure 1A). This increase was partially prevented Orlistat treatment although they do not achieve control values. Pancreatitis results in the accumulation of ascitic fluid containing high amounts of lipase. In ascitic fluid lipase activity was ten fold higher than levels observed in plasma (Figure 1B). As occurs in plasma, Orlistat treatment significantly reduced lipase activity in ascitic fluid. Finally, inflammation in pancreas was characterized by a significant increase in MPO activity (Figure 1C). Lipase inhibition with Orlistat reduced the increases in MPO but differences were not significant.

### Macrophage Activation

**Figure 2:**
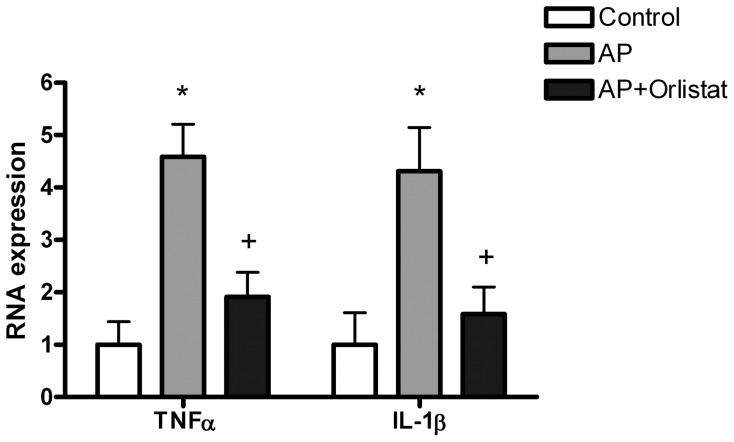
RNA expression of TNFα and IL1β in peritoneal macrophages was significantly induced during acute pancreatitis (AP). Lipase inhibition with Orlistat prevented this activation. *  =  p<0.05 vs control; +  =  p<0.05 vs AP.

Peritoneal macrophages activation during acute pancreatitis was confirmed by the increased mRNA expression of TNFα and IL1β (Figure 2). Inhibition of lipase activity with Orlistat prevented this activation.

### Adipose Tissue Inflammation

**Figure 3:**
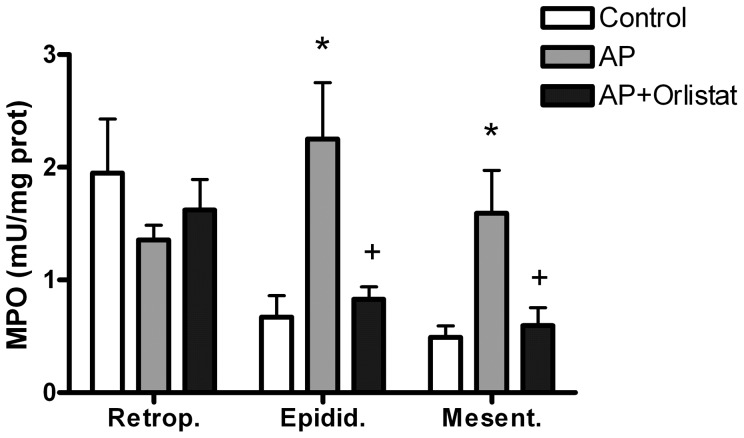
Inflammation in different areas of white adipose tissue was evaluated by measuring myeloperoxidase (MPO) activity. Pancreatitis (AP) results in the induction of MPO activity in epididymal and mesenteric adipose tissue. Lipase inhibition with Orlistat abolished these increases. By contrast, no significant changes were observed in the retroperitoneal adipose tissue. *  =  p<0.05 vs control; +  =  p<0.05 vs AP.

After induction of pancreatitis, patches of fat necrosis were present in mesenteric and perigonadal adipose tissue, but absent in retroperitoneal adipose tissue. Orlistat treatment results in reduced number and extension of fat necrotic areas (data not shown). Pancreatitis also induced significant increases in MPO activity in both epididymal and mesenteric adipose tissue (Figure 3). These increases were completely prevented by Orlistat treatment. By contrast, no changes were observed in MPO activity in retroperitoneal adipose tissue.

### Inflammatory Mediators Expression During Pancreatitis

**Figure 4:**
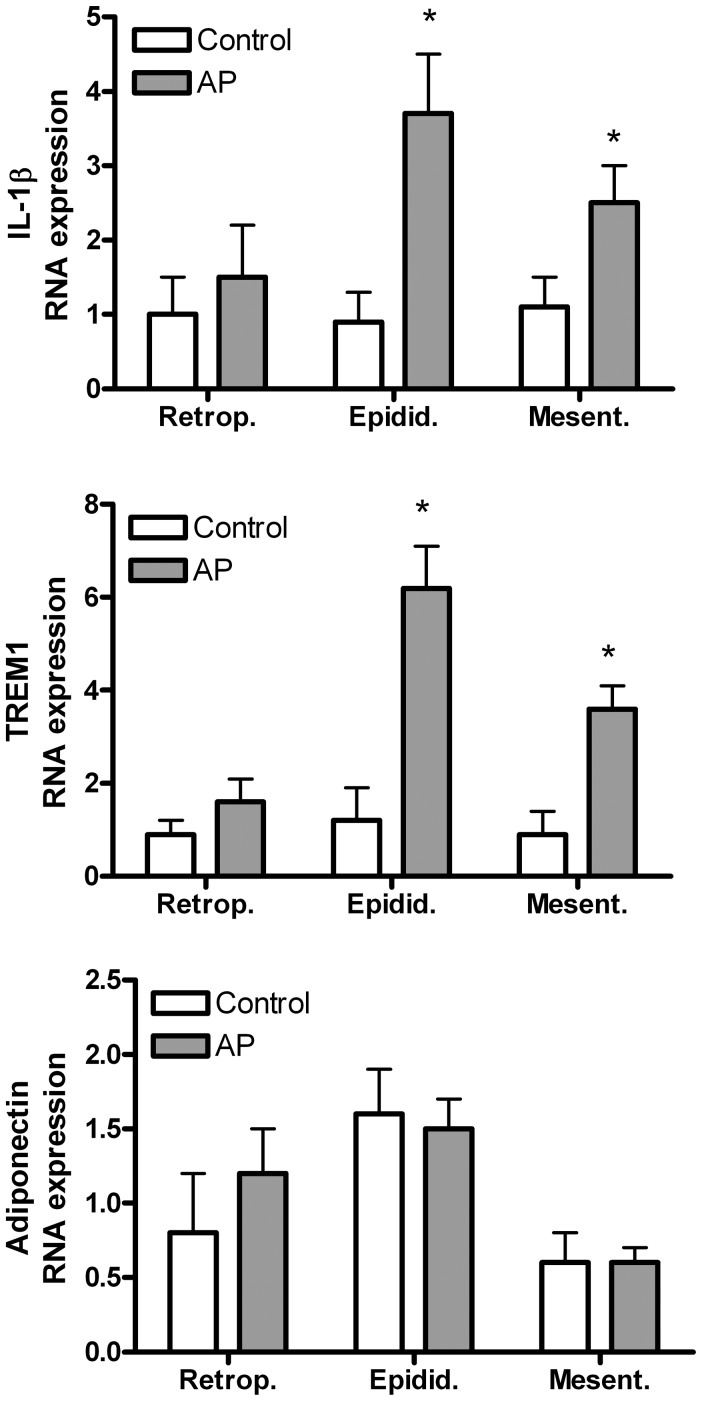
RNA expression of IL1β and TREM1 were induced by acute pancreatitis in epididymal and mesenteric adipose tissues while no changes were observed in retroperitoneal adipose tissue. Adiponectin expression was not induced in any of the three areas of adipose tissue evaluated. *  =  p<0.05 vs control.

When measuring the expression of inflammatory mediators in the different areas of adipose tissue we found that both IL-1β and TREM1 are strongly up-regulated in epididymal and mesenteric adipose tissue (Figure 4). By contrast, no changes were observed in the expression of these mediators on retroperitoneal adipose tissue. On the other hand, expression of adiponectin was not modified in any of the adipose tissues evaluated.

### Histological Analysis

**Figure 5:**
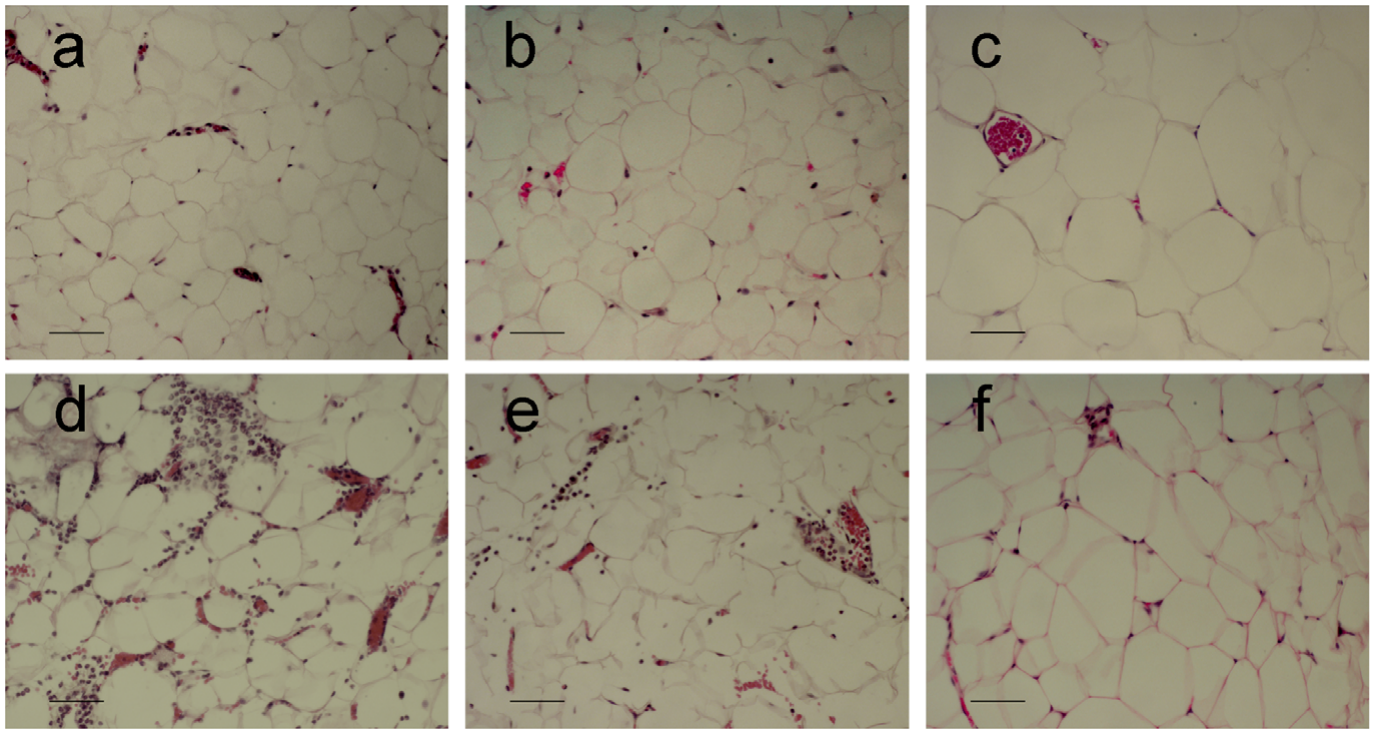
Adipocytes from retroperitoneal areas (c) are higher than that from mesenteric (a) and epididymal (b) regions of adipose tissue. In addition, the number of stromal cells was higher in these regions of adipose tissue. After induction of pancreatitis, an important cell infiltrate could be observed in mesenteric (d) and epididymal (e) areas. By contrast, no relevant infiltrate was observed in retroperitoneal adipose tissue (f).

The main differences observed between the different adipose tissues analyzed were the higher cell size in the adipocytes and a lower number of stromal cells present in the retroperitoneal adipose tissue from control animals (Figure 5). Pancreatitis induces a strong infiltration of polymorphonuclear neutrophils and areas of necrosis in epididymal and mesenteric adipose tissue while no changes could be observed in the retroperitoneal tissue.

### Statistical Analysis

Data have been expressed as mean ± SEM. Means of different groups were compared using a one-way analysis of variance. Tukey’s multiple comparison test was performed for evaluation of significant differences between groups.

## Discussion

White adipose tissue is emerging as an important source of inflammatory mediators in the severe forms of acute pancreatitis [6]–[7], [12]. After the initial damage in the pancreatic gland, cytokines, hydrolytic enzymes and bioactive mediators released by acinar cells act on the adipose tissue inducing necrosis of adipocytes and a strong inflammatory reaction.

The most evident effect is the necrosis of adipocytes by the effect of lipolytic enzymes. These areas of dead tissue promote an intense inflammatory reaction characterized by the infiltration of polymorphonuclear neutrophiles and by the release of important amount of cytokines and other inflammatory mediators. In addition, the release of unsaturated free fatty acids contributes to the extension of cell death and increases the damage to adjacent tissues and the intensity of the local and systemic inflammatory response.

The role of lipotoxicity on the local and systemic inflammation has been demonstrated by Navina et al [7] and our results are in this line. Lipase inhibition with Orlistat significantly reduces tissue inflammation (Figures 1 and 3) but also the activation of peritoneal macrophages (Figure 2). This result confirms that free fatty acids released by the lipolytic activity of pancreatic enzymes plays an important role on the interaction between the adipose tissue and the activation of resident macrophages. Different mechanisms seem to be involved in the proinflammatory effects of fatty acids. Unsaturated fatty acids cause hypocalcemia saponification of peritoneal lipids [13] as well as apoptosis [14]. They could also be chlorinated, by the action of myeloperoxidase, thus generating chlorohydrins with a number of proinflammatory activities [8]. On the other hand, saturated fatty acids serve as ligands for TLR4 expressed in macrophages and induce inflammatory changes through NFkB activation [15].

However, there are significant differences in the response of different adipose tissue sites to acute pancreatitis. Both mesenteric and epididymal adipose tissues showed an intense inflammatory response with infiltration of leukocytes (Figures 3 and 5) and expression of inflammatory mediators (Figure 4). By contrast, no changes were detected on the retroperitoneal adipose tissue.

These three adipose tissues are in the peritoneal cavity of the rats and, during pancreatitis, in direct contact with the ascitic fluid generated by the pancreas. This fluid has a concentration of lipase one order of magnitude higher than plasma (Figure 1), consequently it could be argued that lipolytic activity of ascitic fluid was involved in the triggering of fat necrosis. However, retroperitoneal adipose tissue, that is also in contact with this fluid, does not shown fat necrosis, tissue inflammation or changes in the expression of inflammatory mediators.

When comparing the fatty acid composition of adipose tissue from different sites some differences in the proportion of saturated fatty acids have been observed [16]. Fat intake could also have some influence in the fatty acid composition of adipose tissue [17]. This is of importance since both the toxic and inflammatory effects of fatty acids are very different in function of fatty acid type or level of unsaturation [7]. Therefore it could be expected that the effects of lipase activity on different areas of adipose tissue has different effects on the induction of inflammation and necrosis.

On the other hand, histological analysis revealed that retroperitoneal adipose tissue exhibit remarkable differences with mesenteric and epididymal sites of adipose tissue. In the retroperitoneal area, adipocytes show higher size and there are a lower number of resident macrophages (Figure 5). It is well known that adipose tissue shows important regional differences in metabolic response, but also in immune activity [10], suggesting a different response capacity to stimuli. Macrophages amplify the inflammatory stimulus generating a number of cytokines, chemokines and other metabolites as free radicals or nitric oxide and also shown remarkable heterogeneity between different populations [18], even in the same organ [19]. Some of these cytokines released by activated macrophages, as TNFα, has also the ability to increase the lipolytic activity on adipocytes [15]. Consequently, areas with higher number of resident macrophages could be expected to show a more intense and fast inflammatory response during acute pancreatitis. The possibility that resident macrophages from the different areas of adipose tissue acquired different phenotypes during the progression of acute pancreatitis remains to be investigated.

In acute pancreatitis is still very hard to predict which patients will progress to a severe form of the disease. The magnitude and progression of the systemic inflammatory response does not correlate with the intensity of the initial damage to the pancreas. The heterogeneous response from different adipose tissue sites could help to understand this difficulty. During acute pancreatitis, the adipose tissue modulates the inflammatory response initially triggered in pancreas through releasing free fatty acids, cytokines, chemokines as well as activating leukocytes. The importance of these facts and its effect on the systemic inflammatory response will depend not only on the total amount of adipose tissue affected but also of the particular site of adipose tissue involved in the process. This could be of particular importance in obesity, when the total mass of fat increases significantly. It has been reported that not only the total amount of fat but also its distribution is important on the risk for developing the severe form of the disease [4]–[5].

In conclusion, our results highlight the fact that adipose tissue plays a role in the progression of the systemic inflammation during acute pancreatitis but there is a considerable diversity in different adipose tissue sites. These differences need to be taken into account in order to understand the progression from local pancreatic damage to systemic inflammation during acute pancreatitis and could be important in the final severity of the process.

## Methods

### Animals

Male Wistar rats (250–300 g b.w.) were used in all experiments (Charles River, France). Animals were housed in a controlled environment, fed with standard laboratory pelleted formula (A04, Panlab, Barcelona, Spain) and tap water *ad libitum*. This study conformed to European Community for the use of experimental animals and the institutional committee of animal care and research (C.E.E.A. Univ. Barcelona) approved it.

### Animal Model of Acute Pancreatitis

Animals (n = 6 each group) were anesthetized with an i.p. administration of sodium pentobarbital (50 mg/kg). The biliopancreatic duct was cannulated through the duodenum and the hepatic duct was closed by a small bulldog clamp. Pancreatitis was induced by retrograde perfusion of 5% sodium taurocholate (Sigma, St Louis, Missouri, USA) in a volume of 0.1 ml/100 g b.w. using a perfusion pump (Harvard Instruments, Edenbridge, UK) [11]. To inhibit lipase activity, a group of animals received an i.p. injection of Orlistat (1 ml; 1 mM) (Sigma St Louis, Missouri, USA) at the moment of pancreatitis induction and two more i.p. injections 2 and 4 hours after induction. Control animals received an intraductal perfusion of saline solution (NaCl 0.9%). Six hours after induction the peritoneal macrophages were collected by peritoneal lavage. In addition, samples of plasma, ascitic fluid, pancreas as well as mesenteric, retroperitoneal and perigonadal (epididymal) adipose tissue were obtained, immediately frozen and stored at −80°C until analyzed. Tissue samples were also obtained for histological study.

#### Isolation and culture of macrophages

Peritoneal macrophages were harvested by 5 peritoneal washes with 10 ml of phosphate buffered saline (PBS) containing 3 units/ml heparin. The obtained cell suspension was centrifuged (300×g; 7 min). Cells were suspended in the RPMI1640 culture medium containing 10% fetal calf serum, 2 mM glutamine, penicillin (100 U/ml) and streptomycin (100 µg/ml). Aliquots of about 3×10^6^ cells were plated in 6 wells plates and cultured at 37°C under a gas phase of air/CO_2_ (95:5). After an attachment period of 2 h, the non-adherent cells were removed by shaking. The resulting adherent population consisted of >92% peritoneal macrophages.

### Lipase

Plasma lipase was determined by using commercial turbidimetric assay kits from Randox (Antrim, U.K.), according to the supplier’s specifications.

### Myeloperoxidase

Neutrophilic infiltration was assessed by measuring myeloperoxidase (MPO) activity. MPO was measured photometrically with 3,3′5,5′-tetramethylbenzidine as a substrate. Tissue samples were homogenized with 0.5% hexadecyltrimethylammonium bromide in 50 mM phosphate buffer at pH 6.0. Homogenates were disrupted for 30 seconds using a Labsonic sonicator (Braun Biotech, Inc., Allentown, PA) at 20% power and submitted to three cycles of snap freezing in dry ice and thawing before a final 30 second sonication. Samples were incubated at 60°C for 2 hours and then spun down at 4000×g for 12 minutes. The supernatants were collected for MPO assay. Enzyme activity was assessed photometrically at 630 nm.

### RNA Levels of Inflammatory Mediators

Total RNA from cells or tissue samples were extracted using the TRizol® reagent (Invitrogen, Carlsbad, CA). The RNA was quantified by measurement of the absorbance at 260 and 280 nm using a NanoDrop ND-1000 spectrophotometer (NanoDrop Technologies, USA).

cDNA was synthesized using the iScript cDNA synthesis kit (Bio-Rad Laboratories, Hercules, CA), and reverse transcription was then performed on 1 µg RNA sample by adding iScript reagents. The reaction was incubated at 25°C for 5 min, 42°C for 30 min, and 85°C for 5 min, and then stored at −80°C.

Subsequent PCR amplification was performed in a DNA Engine, Peltier Thermal Cycler (Bio-Rad Laboratories, CA, USA) using IQTM SYBR Green Super mix and the correspondent rat primers: TNFα forward: 5′-AACTCCCAGAAAAGCAAGCA-3′ reverse: 5′-CGAGCAGGAATGAGAAGAGG-3′; IL-1β forward: 5′-AAAAATGCCTCGTGCTGTCT-3′ reverse: 5′-TCGTTGCTTGTCTCTCCTTG-3′; TREM1 forward: 5′-TTTACCATCCTCCGAACGAC-3′ reverse: 5′-CGGGTTGGAGTTGAGTGTTT-3′; Adiponectin forward: 5′-AACTTGTGCAGGTTGGATGG reverse: 5′-CCTGTCATTCCAGCATCTCC-3′; GAPDH forward: 5′-CTGTGTCTTTCCGCTGTTTTC-3′ and reverse: 5′-TGTGCTGTGCTTATGGTCTCA-3′.

Initial denaturation was followed by 40 cycles of DNA amplification with fluorescence detection at the end of the elongation step (SYBRGreen format). Reactions were performed in duplicate and threshold cycle values were normalized to GAPDH gene expression. The specificity of the products was determined by melting curve analysis. The ratio of the relative expression of target genes to GAPDH was calculated by using the ΔC(t) formula.

### Histological Study

For histological studies, tissue samples were fixed in 10% neutral buffered formalin, paraplast-embedded, cut into 5 µm thick sections and stained with hematoxylin–eosin according to standard procedures. Sections were evaluated by light microscopy.
